# The quality of vital signs measurements and value preferences in electronic medical records varies by hospital, specialty, and patient demographics

**DOI:** 10.1038/s41598-023-30691-z

**Published:** 2023-03-08

**Authors:** Niall Jackson, Jessica Woods, Peter Watkinson, Andrew Brent, Tim E. A. Peto, A. Sarah Walker, David W. Eyre

**Affiliations:** 1 Oxford University Hospitals NHS Foundation Trust, Oxford, UK; 2 N Family Club, London, UK; 3 Nuffield Department of Clinical Neurosciences, University of Oxford, Oxford, UK; 4 Nuffield Department of Medicine, University of Oxford, Oxford, UK; 5 NIHR Health Protection Research Unit in Healthcare Associated Infections and Antimicrobial Resistance, University of Oxford, Oxford, UK; 6 Big Data Institute, Nuffield Department of Population Health, University of Oxford, Old Road Campus, Oxford, OX3 7LF UK

**Keywords:** Epidemiology, Diagnostic markers

## Abstract

We aimed to assess the frequency of value preferences in recording of vital signs in electronic healthcare records (EHRs) and associated patient and hospital factors. We used EHR data from Oxford University Hospitals, UK, between 01-January-2016 and 30-June-2019 and a maximum likelihood estimator to determine the prevalence of value preferences in measurements of systolic and diastolic blood pressure (SBP/DBP), heart rate (HR) (readings ending in zero), respiratory rate (multiples of 2 or 4), and temperature (readings of 36.0 °C). We used multivariable logistic regression to investigate associations between value preferences and patient age, sex, ethnicity, deprivation, comorbidities, calendar time, hour of day, days into admission, hospital, day of week and speciality. In 4,375,654 records from 135,173 patients, there was an excess of temperature readings of 36.0 °C above that expected from the underlying distribution that affected 11.3% (95% CI 10.6–12.1%) of measurements, i.e. these observations were likely inappropriately recorded as 36.0 °C instead of the true value. SBP, DBP and HR were rounded to the nearest 10 in 2.2% (1.4–2.8%) and 2.0% (1.3–5.1%) and 2.4% (1.7–3.1%) of measurements. RR was also more commonly recorded as multiples of 2. BP digit preference and an excess of temperature recordings of 36.0 °C were more common in older and male patients, as length of stay increased, following a previous normal set of vital signs and typically more common in medical vs. surgical specialities. Differences were seen between hospitals, however, digit preference reduced over calendar time. Vital signs may not always be accurately documented, and this may vary by patient groups and hospital settings. Allowances and adjustments may be needed in delivering care to patients and in observational analyses and predictive tools using these factors as outcomes or exposures.

## Introduction

Electronic healthcare records (EHR) have been widely adopted across different healthcare settings globally and have become an integral part of health infrastructure: saving time, improving communication and record keeping, and supporting learning^1^. As the scale and breadth of EHR data increases, so does its ability to fulfil secondary functions including quality improvement, product development, and research, contingent on appropriate regulation and transparency^2^. Example applications of EHR data include population-level epidemiological studies^3–5^, machine learning-based diagnostic assistants for clinicians^6^, screening for child maltreatment and family violence^7^, and detecting and tracking infectious disease outbreaks^8,9^.

However, conclusions rely on the reliability and accuracy of EHR data, which is not guaranteed^10,11^. Indeed, the use of EHR data beyond its original purpose (clinical care and billing) raises specific challenges. Data collection in the clinical environment is imperfect^12^ and often incomplete^13^; it may lack comparability or reproducibility^14^ or even simply be wrong^15,16^. Additionally, sensor-derived data such vital signs, are also subject to intrinsic measurement errors arising from variation in calibration, accuracy and drift over time. Attempts to quantify or evaluate EHR data quality are limited, and even fewer have investigated causes for variability in quality^17^. Most studies have focused on checking the accuracy of clinical and diagnostic codes rather than numerical observations. In one notable exception, evaluating the quality of vital sign data across multiple hospitals and EHR systems, there was a skew of completeness and correctness in favour of arriving patients and higher fidelity in wholly EHR based systems compared to a combination of paper and EHR^18^.

Digit preference in vital sign measurement is a well-recognized phenomenon, however it is infrequently formally accounted for in healthcare delivery or in studies using EHR data. Additionally, systems to ensure vital signs are attributable, correct, and contemporaneously recorded can be limited. Many hospitals rely on manual transcription of readings into patient records rather than using potentially more accurate automated systems, e.g. because of cost or problems with interoperability of measurement devices and EHR systems. Terminal digit preference for multiples of ten in blood pressure (BP) recordings has been shown to be extremely common^19,20^, to introduce systemic bias potentially effecting mortality^21^ and to produce inaccurate epidemiological results^22^. This phenomenon has been observed in other vital sign measurements such as respiratory rate^23^, with attempts to rectify inaccuracy through continuous, automated monitoring^24^. Digital preference in vital signs may also impact derived values such as pulse pressure, and platforms that depend on them including early warning systems. However, in addition to standard digit preference, when reviewing data from our hospital group we noted an excess of a specific temperature measurement, 36.0 °C, that appeared unlikely to have arisen from rounding alone, warranting further investigation which we describe here.

We investigated observations of vital signs gathered over 3.5 years from inpatients at a large UK teaching hospital group. We assessed the frequency of recordings with preferences for a specific values (e.g. multiples of ten for BP or temperature readings of 36.0 °C) as a marker of sub-optimal data quality. We extend prior work in this area^19–23^ by investigating associations between digit preference and patient factors, such as age and sex, and hospital factors, such as the specialty caring for a patient. The associations we describe provide insights into what may drive digit preference and may help healthcare institutions improve the quality of the data they collect and use for patient care.

## Methods

### Setting

We conducted a retrospective observational study at Oxford University Hospitals NHS Foundation Trust (OUH) in Oxfordshire, UK. OUH consists of four teaching hospitals with a total of 1000 beds: Hospital A (providing acute care, trauma, and neurosurgery services); Hospital B (elective cancer surgery, transplant, haematology, oncology); Hospital C (district hospital, acute medical services) and Hospital D (elective orthopaedics). OUH acts as a tertiary referral centre for the surrounding region, providing approximately 1 million patient contacts a year and serving a population of around 655,000.

### Data

We used individual observations of vital signs conducted at OUH for adult inpatients (≥ 18y) between 01-January-2016 and 30-June-2019. The vital signs observed, with dates and times of collection, included respiratory rate (RR), heart rate (HR), tympanic temperature, systolic and diastolic BP (SBP and DBP) and oxygen saturations. Vital signs were included for all general wards, but those from intensive care units, operating theatre recovery areas, day case units, and OUH’s hospice were not included as these were collected using a separate system or in locations with a different care delivery focus.

Observations were collected by healthcare assistants and registered nurses using a semi-automated vital sign observation system across all 4 hospital sites. HR, SBP, DBP, and oxygen saturations were collected using an observation machine combining an electronic sphygmomanometer and pulse oximeter. RR was manually timed, typically expected to be recorded by counting the number of breaths over 60 s. Temperature was measured with a separate tympanic thermometer. All observations were then manually transcribed into a tablet computer attached to the same stand, this was usually done at the bedside as the tablet computer allowed the patient’s wristband to be scanned to add results to their record. The tablet computer automatically uploaded results into the EHR in real time. Although the tablet computer and observation equipment were co-located on the same mobile stand, there was no automated check that the observations documented had been performed or matched those measured. We do not believe that any of the measurement devices show any intrinsic value preference. All devices produce an error rather than a default reading if measurement is unsuccessful. Supplemental oxygen devices and alertness (alert, responsive to voice, pain or unresponsive, AVPU) were also recorded. However, these non-numerical measurements are not considered further here. Additional data were obtained: hospital-level data (hospital where the observation was made, the specialty managing the patient); and patient data (age, sex, ethnicity, index of multiple deprivation (IMD) score at home address, Charlson comorbidity score).

### Statistical analysis

Several approaches have been previously described for identifying and quantifying digit preference^25^. For example, jointly estimating a flexible, but smooth, underlying distribution and modelling rounding from adjacent values to the nearest number showing digit preference, e.g. from 9 or 11 to 10^26,27^. Extensions of this approach allow for rounding of groups of adjacent values, e.g. to the nearest 10^28^. However, here we also wanted to account for a phenomenon in temperature recordings which went beyond simple rounding, where a subset of all observations was set to 36.0 °C. We therefore used a simple maximum likelihood-based estimator to jointly estimate the underlying distribution of temperature, HR, SBP, DBP, and respiratory rate measurements, and the proportion of observations affected by digit preference. Oxygen saturation measurements had only limited dynamic range and no clear evidence of digit preference and so were not studied further. For all other vital signs, we assume that a given vital sign follows an underlying distribution, here we fit both normal and gamma distributions. This leads to the following expression for the statistical likelihood of the observed data, given the parameters governing the underlying distribution and any digit preference (i.e. the probability of digit preference and mean/standard deviation or shape/rate):

Pr(observation was subject to digit preference) * Pr(true value is from the interval of the source distribution leading to rounding) + Pr(observation not subject to digit preference) * Pr(true value given the precision values reported at).

In the case of BP and HR recordings, which are initially reported by the measurement device to the nearest whole number, we estimate the extent of rounding to the nearest 10, for example where the BP reading was 120 mmHg, then the likelihood becomes:

Pr(observation rounded) * Pr(observation from the interval [114.5, 124.5)) + Pr(observation not rounded)*Pr(observation from the interval [119.5, 120.5)).

In the case when the HR or BP is not a multiple of ten, then the probability of rounding is set to zero, and only the second term of the likelihood applies. As this term includes the probability that the observation is not rounded it accounts for the fact that rounding leads to depletion in the frequency of observed values relative to the underlying distribution at values that are rounded up/down. The most common way rounding occurs in the RR is by only timing the number of breaths over 15 or 30 s (rather than 60 s), and then multiplying by 4 or 2 respectively to report breaths per minute. We therefore simultaneously estimated the extent of rounding leading to multiples of 4 and 2 for RR. The formula used for the likelihood means that the estimated proportion of observations subject to rounding includes observations where the true value is a multiple of 4 or 2; as such we estimate the total proportion of respiratory rate observations that might have been measured by timing breaths over 15 or 30 s respectively. Similarly, the form of the likelihood for HR and BP rounding means we estimate the total extent of rounding behaviour, including in our calculations the approximately 1 in 10 instances where the true value and the rounded value are the same.

For temperature readings we assume that any true observation can lead to a documented recording of 36.0 °C, as our hypothesis is that an excess of these readings occurs when the temperature is not actually measured but simply documented as 36.0 °C instead, such that the likelihood becomes:

Pr(observation subject to preference for 36.0 °C) + Pr(observation not subject to preference for 36.0 °C) * Pr(observation from the observed interval of the source distribution).

For temperature recordings we make the simplifying assumption that any observation that is not 36.0 °C is not subject to digit preference.

Maximum likelihood estimates were obtained using R, version 4.2 and pnorm, pgamma and optim functions (see Supplement for code). Confidence intervals were estimated by non-parametric bootstrap sampling using 1000 iterations. For computational efficiency only 10,000 observations were included in each iteration. The accuracy of the code was tested through simulation prior to use.

We used multivariable logistic regression to investigate associations between temperature recordings of 36.0 °C and several factors potentially driving value preferences. Analyses were restricted to patients with complete data, and to complete vital sign sets (i.e. all of temperature, HR, RR, SBP, DBP, and oxygen saturations recorded). We used natural cubic splines to account for non-linear relationships for continuous variables (allowing up to five default placed knots, selecting the final number of knots by minimising the Bayesian Information Criterion, BIC). To avoid undue influence of outlying values, continuous variables were truncated at the 1st and 99th percentiles. Pairwise interactions between model main effects were included where this improved model fit based on BIC. We used clustered robust standard errors to account for repeated measurements obtained from the same patient.

To investigate if associations were specific to temperature or applied to vital signs more widely, we also refitted the same model (i.e. with the same spline terms and interactions) with BP digit preference as the outcome, regarding measurements where the SBP and DBP both ended in zero as indicative of possible digit preference. We use this combined measure across both SBP and DBP as it is likely to be most enriched for digit preference. We fitted the same models for HR and RR digit preference regarding readings ending in zero or multiples as two as showing possible digit preference respectively.

We also investigated if the presence of abnormal previous readings affected subsequent digit preference. Temperatures of ≤ 35.5 °C or ≥ 37.5 °C, SBP readings of > 160 or < 90 mmHg, DBP readings of > 100 or < 60 mmHg, HR readings of < 50 or > 120, and RR readings of < 10 and > 24 were arbitrarily considered abnormal. For each observation with a prior observation from the same patient within ≤ 36 h, we selected the most recent prior observation for comparison. A look back period of up to 36 h was allowed to capture vital signs measured just once a day, but at different times. However, where vital signs were measured more frequently only the most recent was considered. We then refitted the regression models above including a term for if the prior vital sign reading (temperature, HR, SBP, DBP, or RR) had been abnormal as a covariate.

Regression analyses were conducted using R, version 4.2.

### Ethical approval

Deidentified data were obtained from the Infections in Oxfordshire Research Database which has approvals from the National Research Ethics Service South Central – Oxford C Research Ethics Committee (19/SC/0403), the Health Research Authority and the national Confidentiality Advisory Group (19/CAG/0144), including provision for use of pseudonymised routinely collected data without individual patient consent. Patients who choose to opt out of their data being used in research are not included in the study. The study was carried out in accordance with all relevant guidelines and regulations.

## Results

Between 01-January-2016 and 30-June-2019, a total of 5,007,650 sets of vital signs were recorded. Of these, 469,904 (9.4%) did not include temperature, 395,445 (7.9%) were missing SBP and/or DBP, 403,364 (8.1%) missing RR, 353,083 (7.1%) missing HR, and 326,474 (6.5%) missing oxygen saturation recordings. Rates of missing data were similar across different patient groups, but missing data were more common near the start of a hospital admission and outside times of day that vital signs were routinely measured. Missing data were more common at Hospital D (elective orthopaedics) and in some specialties, e.g. obstetrics and gynaecology and cardiology (Table S1).

Restricting to complete sets of vital signs left 4,375,654 (87.4%) records in the final dataset from 135,173 patients. The median (IQR) patient age was 61 (42–76) years, 70,515 (52.2%) patients were female, and 100,655 (74.5%) were of white and 27,453 (20.3%) of unstated or unknown ethnicity. The most common specialties recording vital signs were general surgery (882,956, 20.2%), acute and emergency medicine (660,530, 15.1%), and trauma and orthopaedics (597,834, 13.7%).

### Prevalence of value preferences in vital signs readings

**Figure 1:**
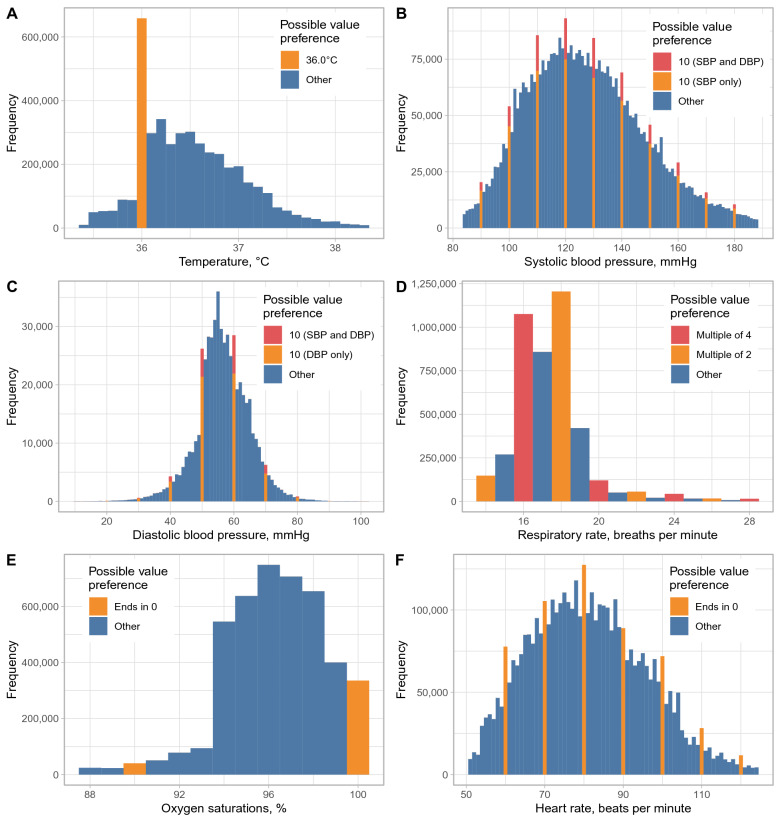
Observed distribution of temperature, systolic blood pressure (SBP), diastolic blood pressure (DBP), respiratory rate, oxygen saturation, and heart rate recordings. Readings showing possible value preferences are shown in orange/red. For SBP and DBP, readings where the SBP and DBP both end in zero are shown in red, readings where only the SBP or DBP respectively end in zero are shown in orange. Values below the 1st percentile or above the 99th percentile are omitted for visualisation purposes.

Compared with the overall distribution of temperature values, there was an excess of temperature readings of 36.0 °C (Fig. 1), readings of 36.0 °C accounted for 15.0% (658,124/4,375,654) of all values. The same pattern of excess readings of 36.0 °C was seen across all four hospitals (Fig. S1). Assuming true temperature readings followed a normal distribution (Table 1, Fig. S2), then 11.3% (95% CI 10.6–12.1%) of observations were estimated to be inappropriately recorded as 36.0 °C instead of the true value. Similar estimates were obtained assuming an alternative gamma distribution for temperature readings (Table S2, Fig. S2).

**Table 1:** Estimated value preference proportions and underlying distributions for temperature, blood pressure, heart rate, and respiratory rate.

Vital sign	Estimated value preference proportion (95% CI)	Estimated mean (95% CI)	Estimated standard deviation (95% CI)
Temperature: Excess of readings of 36.0 °C	11.3% (10.6–12.1%)	36.6 (36.6–36.6)	0.59 (0.58–0.61)
Systolic blood pressure: Readings subject to rounding to nearest 10	2.2% (1.4–2.8%)	128 (127–128)	22.2 (21.9–22.6)
Diastolic blood pressure: Readings subject to rounding to nearest 10	2.0% (1.3–5.1%)	69 (69–70)	12.7 (12.5–14.2)
Heart rate: Readings subject to rounding to nearest 10	2.4% (1.7–3.1%)	81 (81–82)	15.9 (15.6–16.2)
Respiratory rate: Readings subject to rounding to multiples of 2 or 4	Multiple of 2: 22.5% (22.2–24.8%) Multiple of 4: 2.5% (< 0.1–3.5%)	17 (17–18)	2.1 (2.0–2.2)

Model estimates are shown assuming vital signs are normally distributed. Alternative estimates assuming a gamma distribution are shown in Table S2. See Fig. S2 for plots of these distributions with the observed data.

Approximately 1% of all BP readings would be expected to have both a SBP and DBP ending in zero by chance, however 2.3% (99,209) of readings showed this pattern. Assuming SBP and DBP both followed a normal distribution, 2.2% (95% CI 1.4–2.8%) and 2.0% (1.3–5.1%) of readings respectively were estimated to be rounded to the nearest 10 mmHg. Digit preference also occurred for HR readings, with 12.1% (531,219) ending in zero and 2.4% (1.7–3.1%) of readings estimated to be rounded to the nearest ten. RR readings were also more likely to be multiples of 2 (62.0%, 2,711,524) or 4 (29.1%, 1,273,972) than expected by chance, with digit preference to the nearest multiple of 2 or 4 affecting an estimated 22.5% (22.2–24.8%) and 2.5% (< 0.1–3.5%) of readings respectively. Estimates were similar if SBP, DBP, HR, and RR were assumed to be gamma distributed, with the exception that rounding of RR to the nearest multiple of 4 was found to be less common, < 0.1% (< 0.1–0.1%) (Table S2). There was no clear evidence of value preference in oxygen saturation readings (Fig. 1).

### Value preference associations with patient demographics

**Figure 2:**
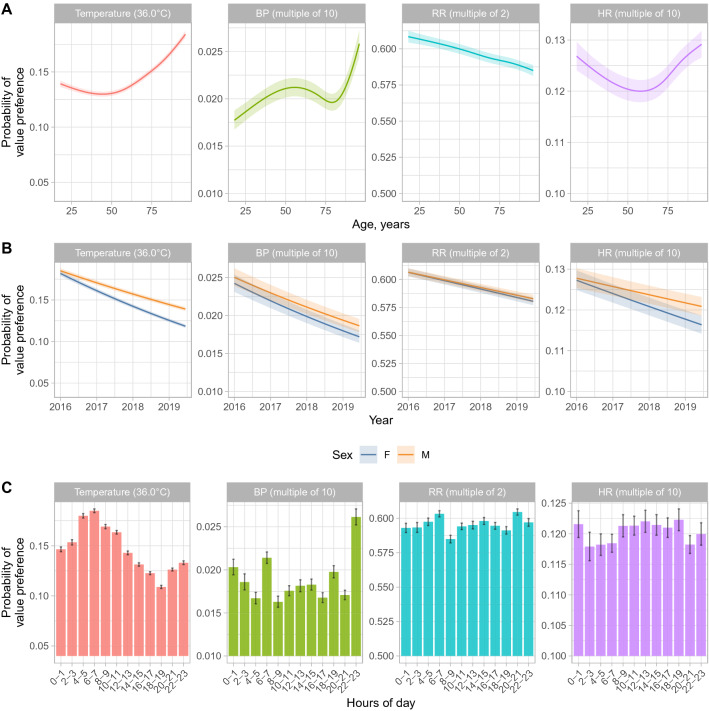
Multivariable associations between age, study year, sex, and time of day and vital sign value preferences. Model predictions from a multivariable model are shown with all other factors set to the reference category or median value as shown in Table 3 and Fig. 3. The shaded ribbon indicates the 95% confidence interval. *BP* blood pressure, *RR* respiratory rate, *HR* heart rate, *M* male, *F* female. The minimum value on each y-axis represents the approximate expected value without any value preference.

**Figure 3:**
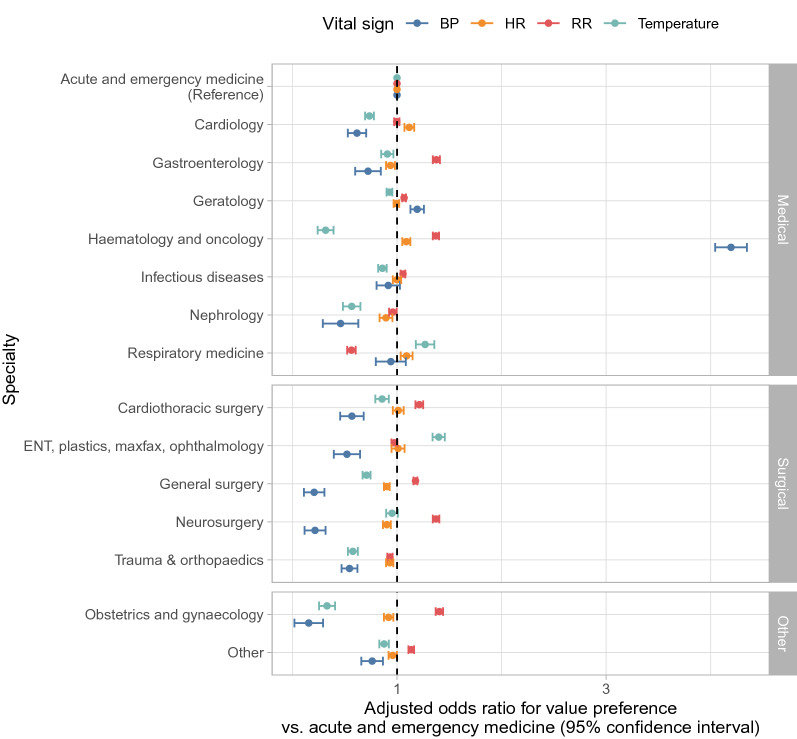
Relationship between specialty and vital sign value preferences. *ENT* ear, nose and throat, *plastics* plastic surgery, *maxfax* maxillofacial surgery, *BP* blood pressure, *RR* respiratory rate, *HR* heart rate. Values plotted are provided in Table S3.

Associations with value preferences for each vital sign were investigated using multivariable models (Tables 2, 3 and Figs. 2, 3). For 41,350 (0.9%) records no deprivation score was documented; these records were excluded. Complete data were available for all other hospital/patient variables. Temperature was independently more likely to be recorded as 36.0 °C with increasing age above 50 years and BP most likely to be recorded with SBP and DBP both ending in zero for those above 80 years (Fig. 2A). Conversely, RR was less likely to be a multiple of 2 as age increased and HR value preference was greatest in younger and older adults. Male patients were more likely than female patients to have readings with value preferences across all vital signs, with differences by sex increasing for temperature and HR as performance improved overall with passing calendar time (Fig. 2B). Temperature value preference was slightly less common in patients from less deprived areas (aOR per 10 unit change in deprivation percentile = 0.99 [0.99–0.99, higher percentiles are less deprived]), but with no evidence of a difference in other vital signs. There was no evidence for consistent differences in value preference by ethnicity, however temperatures of 36.0 °C were more commonly recorded in patients of Asian ethnicity (aOR vs. white = 1.08 [1.03–1.13]) and BPs ending in zero were more common in those of unstated or unknown ethnicity (aOR vs. white = 1.13 [1.07–1.19]). Patients with higher Charlson scores were slightly more likely to have recorded temperatures of 36.0 °C (aOR per 5 unit increase = 1.02 [1.02–1.03]), but less likely to have BP ending in zero (aOR per 5 unit increase = 0.93 [0.92–0.94]), with only small changes by Charlson score in HR or RR value preferences.

**Table 2:** Value preferences in temperature, blood pressure, respiratory rate and heart rate and descriptive data for associated factors.

Characteristic	Temperature		Systolic and diastolic blood pressure		Respiratory rate		Heart rate	
	Not 36.0 °C	36.0 °C	Not ending in 0	Both end in 0	Not multiple of 2	Multiple of 2	Not ending in 10	Ends in 10
	N = 3,717,530	N = 658,124	N = 4,276,445	N = 99,209	N = 1,664,130	N = 2,711,524	N = 3,844,435	N = 531,219
Age	70 (55, 82)	74 (59, 84)	71 (55, 82)	67 (54, 80)	71 (56, 82)	71 (55, 82)	71 (55, 82)	71 (56, 82)
Sex								
F	1,859,740 (85%)	316,025 (15%)	2,129,244 (98%)	46,521 (2.1%)	827,373 (38%)	1,348,392 (62%)	1,914,306 (88%)	261,459 (12%)
M	1,857,790 (84%)	342,099 (16%)	2,147,201 (98%)	52,688 (2.4%)	836,757 (38%)	1,363,132 (62%)	1,930,129 (88%)	269,760 (12%)
Hospital								
A	2,167,583 (85%)	392,533 (15%)	2,519,249 (98%)	40,867 (1.6%)	993,501 (39%)	1,566,615 (61%)	2,247,311 (88%)	312,805 (12%)
B	850,253 (87%)	122,539 (13%)	927,208 (95%)	45,584 (4.7%)	363,946 (37%)	608,846 (63%)	856,875 (88%)	115,917 (12%)
C	378,039 (78%)	104,115 (22%)	474,505 (98%)	7649 (1.6%)	167,796 (35%)	314,358 (65%)	422,181 (88%)	59,973 (12%)
D	321,655 (89%)	38,937 (11%)	355,483 (99%)	5109 (1.4%)	138,887 (39%)	221,705 (61%)	318,068 (88%)	42,524 (12%)
IMD percentile, higher values are less deprived	74 (54, 89)	72 (53, 89)	74 (54, 89)	74 (55, 90)	74 (54, 89)	74 (54, 89)	74 (54, 89)	73 (54, 89)
Unknown IMD percentile	35,791	5559	40,372	978	15,806	25,544	36,500	4850
Ethnic group								
White	2,917,017 (85%)	529,410 (15%)	3,373,641 (98%)	72,786 (2.1%)	1,315,531 (38%)	2,130,896 (62%)	3,026,375 (88%)	420,052 (12%)
Asian	80,345 (85%)	13,902 (15%)	91,856 (97%)	2391 (2.5%)	35,493 (38%)	58,754 (62%)	82,748 (88%)	11,499 (12%)
Black	41,091 (86%)	6431 (14%)	46,069 (97%)	1453 (3.1%)	17,625 (37%)	29,897 (63%)	41,787 (88%)	5735 (12%)
Mixed	21,152 (86%)	3348 (14%)	23,856 (97%)	644 (2.6%)	9044 (37%)	15,456 (63%)	21,510 (88%)	2990 (12%)
Other	26,168 (87%)	4009 (13%)	29,589 (98%)	588 (1.9%)	11,346 (38%)	18,831 (62%)	26,569 (88%)	3608 (12%)
Ethnicity not stated or unknown	631,757 (86%)	101,024 (14%)	711,434 (97%)	21,347 (2.9%)	275,091 (38%)	457,690 (62%)	645,446 (88%)	87,335 (12%)
Charlson score	4 (0, 14)	5 (0, 14)	4 (0, 14)	5 (0, 13)	4 (0, 14)	4 (0, 14)	4 (0, 14)	4 (0, 14)
Day of admission	4 (1, 11)	5 (1, 13)	4 (1, 11)	6 (2, 15)	4 (1, 11)	4 (1, 12)	4 (1, 11)	4 (1, 12)
Hour of day	13 (7, 19)	11 (6, 17)	13 (7, 19)	13 (7, 19)	13 (7, 18)	13 (7, 19)	13 (7, 19)	13 (7, 18)
Day of the week								
Monday	497,949 (85%)	90,130 (15%)	574,066 (98%)	14,013 (2.4%)	223,895 (38%)	364,184 (62%)	516,337 (88%)	71,742 (12%)
Tuesday	523,487 (85%)	93,329 (15%)	602,258 (98%)	14,558 (2.4%)	233,833 (38%)	382,983 (62%)	541,922 (88%)	74,894 (12%)
Wednesday	541,697 (85%)	95,465 (15%)	623,070 (98%)	14,092 (2.2%)	243,970 (38%)	393,192 (62%)	560,120 (88%)	77,042 (12%)
Thursday	545,822 (85%)	95,728 (15%)	627,541 (98%)	14,009 (2.2%)	246,589 (38%)	394,961 (62%)	563,769 (88%)	77,781 (12%)
Friday	551,709 (85%)	96,725 (15%)	634,780 (98%)	13,654 (2.1%)	246,326 (38%)	402,108 (62%)	569,959 (88%)	78,475 (12%)
Saturday	544,593 (85%)	95,648 (15%)	625,791 (98%)	14,450 (2.3%)	242,518 (38%)	397,723 (62%)	562,806 (88%)	77,435 (12%)
Sunday	512,273 (85%)	91,099 (15%)	588,939 (98%)	14,433 (2.4%)	226,999 (38%)	376,373 (62%)	529,522 (88%)	73,850 (12%)
Years since study start	1.80 (0.96, 2.67)	1.64 (0.85, 2.46)	1.78 (0.95, 2.64)	1.56 (0.77, 2.52)	1.80 (0.97, 2.66)	1.76 (0.93, 2.63)	1.78 (0.95, 2.64)	1.74 (0.92, 2.62)
Specialty group								
Acute & emergency medicine	540,795 (82%)	119,735 (18%)	648,723 (98%)	11,807 (1.8%)	259,057 (39%)	401,473 (61%)	578,130 (88%)	82,400 (12%)
General surgery	766,553 (87%)	116,403 (13%)	870,928 (99%)	12,028 (1.4%)	332,571 (38%)	550,385 (62%)	780,674 (88%)	102,282 (12%)
Cardiothoracic surgery	92,115 (85%)	16,121 (15%)	106,641 (99%)	1595 (1.5%)	40,270 (37%)	67,966 (63%)	94,940 (88%)	13,296 (12%)
ENT, plastic surgery, maxillofacial surgery, ophthalmology	130,533 (82%)	28,216 (18%)	156,212 (98%)	2537 (1.6%)	63,464 (40%)	95,285 (60%)	139,468 (88%)	19,281 (12%)
Neurosurgery	178,643 (85%)	30,416 (15%)	206,346 (99%)	2713 (1.3%)	72,799 (35%)	136,260 (65%)	184,818 (88%)	24,241 (12%)
Trauma & orthopaedics	522,560 (87%)	75,274 (13%)	589,144 (99%)	8690 (1.5%)	234,405 (39%)	363,429 (61%)	526,790 (88%)	71,044 (12%)
Obstetrics & gynaecology	94,109 (91%)	9,839 (9.5%)	102,672 (99%)	1276 (1.2%)	35,843 (34%)	68,105 (66%)	92,079 (89%)	11,869 (11%)
Cardiology	160,615 (84%)	31,405 (16%)	189,256 (99%)	2764 (1.4%)	74,350 (39%)	117,670 (61%)	166,826 (87%)	25,194 (13%)
Gastroenterology	105,449 (82%)	23,572 (18%)	127,042 (98%)	1979 (1.5%)	42,575 (33%)	86,446 (67%)	113,514 (88%)	15,507 (12%)
Geratology	377,153 (82%)	82,552 (18%)	450,489 (98%)	9216 (2.0%)	177,581 (39%)	282,124 (61%)	401,963 (87%)	57,742 (13%)
Infectious diseases	190,250 (83%)	39,638 (17%)	225,820 (98%)	4068 (1.8%)	87,968 (38%)	141,920 (62%)	201,193 (88%)	28,695 (12%)
Nephrology	75,973 (87%)	11,189 (13%)	85,818 (98%)	1344 (1.5%)	35,481 (41%)	51,681 (59%)	77,003 (88%)	10,159 (12%)
Haematology & oncology	264,698 (89%)	31,497 (11%)	261,407 (88%)	34,788 (12%)	104,437 (35%)	191,758 (65%)	258,857 (87%)	37,338 (13%)
Respiratory medicine	57,624 (82%)	12,849 (18%)	69,228 (98%)	1245 (1.8%)	32,109 (46%)	38,364 (54%)	61,367 (87%)	9,106 (13%)
Other	160,460 (85%)	29,418 (15%)	186,719 (98%)	3159 (1.7%)	71,220 (38%)	118,658 (62%)	166,813 (88%)	23,065 (12%)

Data for patient factors (age, sex, ethnicity, Index of multiple deprivation, Charlson score), are summarised per observation, rather than per patient. Therefore, for example as older patients had longer hospital stays and more observations the median age is higher than the median age on a per patient basis. *ENT* ear, nose and throat surgery. Data are summarised as median (interquartile range) or n (%). Hospital A provides acute care, trauma, and neurosurgery services, hospital B provides elective cancer surgery, transplant, haematology, oncology services, hospital C is a district hospital providing acute care and hospital D is an elective orthopaedic hospital.

**Table 3:** Multivariable relationships between value preferences in temperature, blood pressure, respiratory rate and heart rate and associated factors.

Characteristic	Temperature			Systolic and diastolic blood pressure			Respiratory rate			Heart rate		
	aOR	95% CI	p-value	aOR	95% CI	p-value	aOR	95% CI	p-value	aOR	95% CI	p-value
Hospital												
A	–	–		–	–		–	–		–	–	
B	0.93	0.91, 0.95	< 0.001	1.19	1.12, 1.27	< 0.001	0.98	0.97, 0.99	0.001	0.98	0.96, 0.99	< 0.001
C	1.38	1.35, 1.41	< 0.001	0.91	0.88, 0.94	< 0.001	1.23	1.21, 1.24	< 0.001	0.99	0.97, 1.01	0.22
D	0.78	0.76, 0.80	< 0.001	0.90	0.86, 0.94	< 0.001	1.08	1.07, 1.10	< 0.001	0.99	0.97, 1.01	0.47
IMD percentile, per 10% less deprived	0.99	0.99, 0.99	< 0.001	1.00	0.99, 1.01	0.94	1.00	1.00, 1.00	0.071	1.00	1.00, 1.00	0.13
Ethnic group												
White	–	–		–	–		–	–		–	–	
Asian	1.08	1.03, 1.13	0.003	1.11	0.95, 1.29	0.19	1.01	0.99, 1.03	0.50	1.01	0.98, 1.05	0.46
Black	1.00	0.93, 1.07	0.89	1.17	0.95, 1.45	0.13	1.03	1.00, 1.07	0.082	1.01	0.95, 1.08	0.77
Mixed	1.01	0.91, 1.11	0.92	1.16	0.81, 1.66	0.43	1.03	0.99, 1.06	0.20	1.01	0.96, 1.07	0.60
Other	0.95	0.88, 1.02	0.17	0.94	0.82, 1.07	0.34	1.00	0.97, 1.04	0.79	1.00	0.95, 1.04	0.90
Ethnicity not stated or unknown	0.98	0.96, 1.00	0.022	1.13	1.07, 1.19	< 0.001	1.02	1.01, 1.02	< 0.001	0.99	0.98, 1.00	0.049
Charlson score, per 5 unit change	1.02	1.02, 1.03	< 0.001	0.93	0.92, 0.94	< 0.001	1.00	0.99, 1.00	< 0.001	1.01	1.01, 1.01	< 0.001
Day of admission, per 7 day change	1.03	1.03, 1.03	< 0.001	1.04	1.03, 1.05	< 0.001	1.01	1.00, 1.01	< 0.001	1.01	1.00, 1.01	< 0.001
Day of the week												
Monday	–	–		–	–		–	–		–	–	
Tuesday	0.99	0.98, 1.01	0.31	0.99	0.96, 1.01	0.3	1.01	1.00, 1.01	0.12	1.00	0.98, 1.01	0.42
Wednesday	0.99	0.98, 1.00	0.13	0.91	0.89, 0.94	< 0.001	0.99	0.98, 1.00	0.009	0.99	0.98, 1.00	0.17
Thursday	0.99	0.98, 1.00	0.20	0.90	0.87, 0.92	< 0.001	0.98	0.98, 0.99	< 0.001	1.00	0.99, 1.01	0.50
Friday	1.00	0.99, 1.01	0.71	0.87	0.85, 0.89	< 0.001	1.00	1.00, 1.01	0.47	0.99	0.98, 1.01	0.33
Saturday	1.00	0.98, 1.01	0.34	0.94	0.92, 0.97	< 0.001	1.01	1.00, 1.02	0.063	0.99	0.98, 1.01	0.29
Sunday	1.00	0.98, 1.01	0.34	1.01	0.98, 1.03	0.66	1.02	1.01, 1.03	< 0.001	1.01	1.00, 1.02	0.29

Odds ratios above 1 indicate that value preference is more common than in the reference group. Adjustment is also made for age, sex, study year, and time of day (shown in Fig. 2), and relationships with the specialty caring for the patient (Fig. 3). Hospital A provides acute care, trauma, and neurosurgery services, hospital B provides elective cancer surgery, transplant, haematology, oncology services, hospital C is a district hospital providing acute care and hospital D is an elective orthopaedic hospital.

*aOR* adjusted odds ratio, *CI* confidence interval.

### Changes over calendar time, hour of day, and by hospital and time in admission

The frequency of value preferences for all vital signs decreased during the study (Fig. 2B). Temperatures were most likely to be recorded as 36.0 °C at around 6-8am, i.e. at the first routine set of observations performed per day in most patients, whereas BP was most likely to end in zero during the late evening, and to a lesser extent between 6 and 8am. Relatively little change in HR or RR value preference was seen by time of day (Fig. 2C). Value preference for all vital signs became slightly more common the greater the prior length of stay (e.g. for temperature, aOR per 7 day increase in prior length of stay = 1.03 [95% CI 1.03–1.03]).

Differences were also seen between hospitals in temperature value preferences. After adjusting for differences in the specialties present and all other factors, compared to hospital A (acute care, trauma, and neurosurgery), value preferences in temperature measurement were more common in hospital C (district hospital) and less common in hospital B (elective cancer surgery, transplant, haematology, oncology) and particularly hospital D (elective orthopaedics) (Table 3). RR value preference was also more common in hospital C, as well as D, whereas BP value preference was more common in hospital B. Relatively little difference between hospitals was seen in HR value preference.

### Variation by specialty

Value preferences in temperature, BP, and RR readings varied by specialty, whereas differences in HR value preference were more limited (Fig. 3). Compared to acute and emergency medicine, value preferences for temperature and BP were less common in most surgical specialties and medical sub-specialties, but RR value preference was more common in several surgical specialties and BP value preferences substantially more common in haematology and oncology. Value preference in temperature readings was least common in haematology and oncology. Both nephrology and trauma and orthopaedics exhibited less value preference across all vital signs than acute and emergency medicine.

### Effect of previous abnormal measurements

A total of 4,125,851 sets of observations had a previous measurement from the same patient within  ≤ 36 h. Temperature readings of 36.0 °C were less frequent following an abnormal prior temperature measurement, 20,224/354,837 (5.7%), than following a normal prior measurement, 601,092/3,771,014 (16.0%). Given it may take time for temperature to normalise it would not be expected that those with a previously abnormal temperature would have the same proportion of true temperatures of 36.0 °C as the overall hospital population (estimated as 4.0% from the normal distribution fitted above). However even allowing for this, preference for recording a temperature of 36.0 °C was likely more common with a normal prior measurement. Adjusting for the same factors as in the main analysis, a previous abnormal temperature independently reduced the odds of a recording of 36.0 °C (aOR = 0.34, [95% CI 0.34–0.35]). Similarly, 25,091/1,307,048 (1.9%) of BP observations had SBP and DBP both ending in zero after an abnormal SBP or DBP, compared to 69,773/2,818,803 (2.5%) without a prior abnormal reading (aOR = 0.86 [0.84–0.88]). However, the opposite pattern was seen for HR and RR where abnormal previous readings were associated with increased subsequent digit preference (aOR = 1.22 [1.19–1.24] and aOR = 1.12 [1.10–1.14] respectively).

## Discussion

In this analysis of records from a large UK teaching hospital group, we show preference for specific values or digits in vital sign records in EHRs. Our findings have implications for patient management, quality improvement initiatives and for research conducted using EHRs. Three potential mechanisms underlie the value preferences seen. HR and BP measurements exhibit classical digit preference with rounding occurring during human transcription of readings. Value preference in RR most likely occurred during the measurement process, with RR values that are multiples of two arising from recording the number of breaths per minute over 30 s and doubling the measured count. Thirdly, value preferences in temperature readings occurred due to preference for a specific value, 36.0 °C.

A key question is whether temperature value preferences indicate simply convenience rounding on transcribing values or whether they are also a marker for incompletely observed observations. Potentially favouring the latter, we observed differences in the relative frequency of value preferences. We found that around 2% of BP and HR measurements showed evidence of rounding to the nearest 10. In contrast, over 5-times more temperature readings were estimated to affected by value preference: an estimated excess of 11% of all temperature recordings were recorded as 36.0 °C. One alternative explanation to rounding is that these recordings were documented when in fact no temperature was measured, e.g. it was presumed to be normal where the thermometer, which was separate to the rest of the vital sign measuring equipment, was missing or not working, or alternatively where patients appeared well and a normal measurement was assumed as has been hypothesised for respiratory rate measurement too^29^. It is also possible that implausibly low readings, e.g. when thermometers were mis-calibrated, were recorded as 36.0 °C, but this is unlikely to have been common. Although over 20% of RR recordings were estimated to be rounded to the nearest two, this most likely reflects the measurements process described above, rather than digit preference per se. Automated measurements of RR may increase accuracy, depending on the setting and device used, in some instances automated RR measurements have been shown correlated better with outcomes^30^, but not in others^29^.

We found differences between hospital specialties, even after adjustment for other factors. Generally surgical specialities recorded vital signs with greater precision than acute and emergency medicine. However, the prevalence of value preferences also potentially reflects the culture within a speciality, where greater importance may be placed on measured values, e.g. in nephrology, or on specific vital signs, e.g. temperature in neutropenic and other immunosuppressed patients in haematology and oncology or BP in cardiology. We also found marked differences in temperature measurement between the four hospitals in the organisation, even following adjustment for the specialties present. This may reflect systemic factors, e.g. staffing levels and the importance placed on vital signs may vary by setting. In higher acuity settings, reliance on vital signs for treatment escalation could increase vital sign fidelity compared to less acute settings focused on rehabilitation. Although patients are admitted to acute medicine as an emergency, the increased digit preference seen in this specialty may reflect that for many longer staying patients rehabilitation and provision of social care are the dominant issues for much of each admission. We also found that normal prior temperature and BP measurements were more likely to be followed by digital preference in subsequent observations, with previous abnormal measurements being associated with greater accuracy in subsequent observations. However, this effect was not seen consistently with the opposite for HR and RR, where possibly 3 digit heart rates are more likely to be rounded or more rapid respiratory rates more difficult to count precisely.

Older patients and male patients were more likely to have temperature and BP recordings with value preferences, whereas RR value preferences we more common in younger patients. Further work is required to better understand the reasons for this. For example, variation in temperature recording by age may reflect differences in the acuity of patients and associated culture around vital sign measurement, the relative importance placed on curative treatment vs. patient comfort, and physical barriers to temperature measurement including patient agitation. There were no systematic differences by ethnicity across all vital signs.

Changes over time suggest institution-wide improvement is possible, with increased precision of all vital signs seen during the study. The study builds on previous studies of vital sign recording quality^31^, and highlights that institutions may wish to monitor vital sign recording to identify areas of the hospital or patient groups where specific interventions to improve quality may be required.

Multiple variables representing the timing of measurements were investigated. Routine morning temperature measurements, e.g. 6–7am, were most likely to be impacted by digit preference. BP measurements were also more likely to be rounded in morning as well as in late evening. Vital sign precision was greatest around the time of hospital admission with value preferences increasing as length of stay increased, likely reflecting that patients are most unwell when first presenting to hospital and so vital signs are performed and recorded carefully. However, it was also more common to have one or more vital signs missing after short prior lengths of stay, e.g. < 1 day, possibly reflecting different approaches to short stay patients, or rechecking of specific vital signs in some acute settings. There was less temporal variation in digit preference in RR and HR measurements.

Digit preference is a well described phenomenon^19,20^. However, particularly for temperature measurement, the question that arises from our findings is; if a vital sign is more difficult to measure for some reason, then why does current culture potentially favour documenting an inaccurate reading instead of leaving it missing, especially within a system where safety is prioritised. There may be explicit or implied pressure to always record a complete set of vital signs but less scrutiny of their accuracy^32^ (although ~ 13% of observations in our study were excluded because of missing one or more vital signs), or it may be that recording an observation as unavailable may be more onerous and require entering a justification. There may also be disincentives to recording abnormal values if this requires escalation of care and additional action. Related to this point, value preferences may impact early warning scores, such as NEWS2^33^, e.g. value preferences for temperatures of 36.0 °C may score a point that would not otherwise be scored with temperatures ranging from 36.1 to 38.0 °C.

Limitations of our study include that it is based on a single organisation and data entry system for recording vital signs. Further studies are required to confirm if our findings are replicated more widely. As this was a retrospective study, we were not able to identify the reasons behind missing or potential inaccurate readings; future investigations could consider both practical barriers such as malfunctioning devices and behavioural factors such as perceptions around the importance of vital signs. We did not investigate more granular variation in vital sign recording by hospital ward or individual staff member, the latter as the identity of the healthcare worker recording the vital signs was not available in our data extract. We also did not investigate the downstream consequences of vital sign values (as has been done elsewhere to create early warning systems) or the consequences of value preferences. The latter could be looked at in future work, e.g. considering associations with length of stay or mortality, although care would be required to avoid reverse causation where delays in discharge or a more palliative focus change value preferences.

There are also several technical limitations. Our model for estimating the proportion of vital signs affected by value preference is relatively simple. For BP and HR, we only consider rounding to the nearest 10, which was the most dominant form in our data, but rounding to the nearest 5 or 2 also occurs. However, our main focus here is not the absolute quantification of value preference, but rather to explore the potential drivers of it and to highlight it as an issue. Our estimation framework could be extended to consider multiple types of rounding, e.g. by expanding the likelihood to simultaneously consider rounding to the nearest 2, 5 and 10. We also assume that all underlying temperatures are equally likely to be recorded as 36.0 °C; in reality external signs of a fever, which is often accompanied by other abnormal vital signs, may prompt more accurate recording of the temperature. The underlying distributions chosen result in a good fit for HR and BP, particularly the gamma distribution. For temperature the fit is less good, but a reasonable approximation and a better fitting distribution is unlikely to explain the substantial excess in recordings of 36.0 °C. There are relatively few unique commonly recorded RR values resulting in the fitted continuous distribution being a less good approximation. The logistic regression models fitted include both true values and recordings affected by value preferences as outcomes. Therefore, for temperature where an absolute value preference is common, it is possible that in part the resulting associations are indicative of a normal temperature of 36.0 °C as well as value preferences. For all other vital signs value preferences occur throughout the full range of measurements and so the logistic models are still able to estimate factors associated with a relative increase in value preferences robustly. Finally, inaccuracies not leading to value preferences are not assessed in the current analysis, but also need to be considered when using EHR data, e.g. miscalibration of devices or measurement error arising from failure of tympanic thermometers to accurately record low temperatures.

Our study provides evidence that vital sign measurement displays value preference to a such a degree that it could affect conclusions based on unadjusted vital sign data, in both clinical and research settings. We show that hospital, speciality, admission stage and patient age all have important impacts on the accuracy of vital signs. Changes over time in our hospital suggest improvements in accuracy are possible. Ultimately fully connected systems that automatically measure and/or record vital signs into patient records are likely to address many of the issues identified; however, these are only likely to be implemented if this is prioritised by device manufacturers and healthcare providers. Work with institutions and individuals is required to fully elucidate and understand the mechanisms behind values preferences on a systems, patient and clinician level. Greater consensus on what health information is essential and what level of accuracy is required, across different settings, would help define benchmarks for acceptable performance, which could potentially be monitored automatically. In the meantime, clinicians and researchers need to be aware that vital signs may not always be accurately documented, and to make appropriate allowances and adjustments for this in delivering care to patients and in analyses using these factors as outcomes or exposures.

## Supplementary Information

Supplementary Information.

## Data Availability

The data analysed are not publicly available as they contain personal data but are available from the Infections in Oxfordshire Research Database (https://oxfordbrc.nihr.ac.uk/research-themes-overview/antimicrobial-resistance-and-modernising-microbiology/infections-in-oxfordshire-research-database-iord/), subject to an application and research proposal meeting on the ethical and governance requirements of the Database. More information is available by emailing iord@ndm.ox.ac.uk.
