# Supplementary material for: The quality of vital signs measurements and value preferences in electronic medical records varies by hospital, specialty, and patient demographics
Source: Sci Rep. 2023 Mar 8;13:3858. doi: 10.1038/s41598-023-30691-z (PMC9995491; doi:10.1038/s41598-023-30691-z)
Supplement: Supplementary file 1 — Supplementary Information. [file 41598_2023_30691_MOESM1_ESM.pdf]

The quality of vital signs measurements and value preferences in electronic medical records varies by hospital, specialty, and patient demographics: Supplementary material

Supplementary methods: R code

Likelihood function for temperature (normal distribution). Generate maximum likelihood estimates and return estimates on original scale, where df$temp is a vector of temperature readings recorded to 1 decimal place:

```
ll = function(par, x) {
  mean = exp(par[1])
  sd = exp(par[2])
  p_fixed = plogis(par[3])

  liki = log( p_fixed * (x==36) +
              (1-p_fixed) * ( pnorm(x+0.05, mean, sd) -
                              pnorm(x-0.05, mean, sd) )
            )
  return (-sum(liki))
}

o = optim(par=c(log(30),1,0.1), ll, x=df$temp)
exp(o$par[1])
exp(o$par[2])
plogis(o$par[3])
```

Likelihood function for temperature (gamma distribution). Generate maximum likelihood estimates and return estimates on original scale, where df$temp is a vector of temperature readings recorded to 1 decimal place:

```
ll_gamma = function(par, x) {
  shape = exp(par[1])
  rate = exp(par[2])
  p_fixed = plogis(par[3])

  liki = log( p_fixed * (x==36) +
                (1-p_fixed) * (pgamma(x+0.05, shape=shape, rate=rate) -
                       pgamma(x-0.05, shape=shape, rate=rate) )
                )
  return (-sum(liki))
}

o = optim(par=c(log(3.5),log(0.1),1), ll_gamma, x=df$temp)
exp(o$par[1])
exp(o$par[2])
plogis(o$par[3])
```

Likelihood function for blood pressure and heart rate (normal distribution). Generate maximum likelihood estimates and return estimates on original scale, where df$bp is a vector of systolic blood pressure, diastolic blood pressure or heart rate readings recorded as integers:

```
ll_bp = function(par, x) {
  mean = exp(par[1])
  sd = exp(par[2])
  p_round = plogis(par[3])

  liki = log( p_round *(x %% 10 == 0) *
                (pnorm(x+4.5, mean, sd) -  pnorm(x-5.5, mean, sd)) +
            (1-p_round) *
                (pnorm(x+0.5, mean, sd) -  pnorm(x-0.5, mean, sd) )
           )
  return (-sum(liki))
}

o = optim(par=c(log(120),log(20),-3), ll_bp, x=df$bp)
exp(o$par[1])
exp(o$par[2])
plogis(o$par[3])
```

Likelihood function for blood pressure and heart rate (gamma distribution). Generate maximum likelihood estimates and return estimates on original scale, where df$bp is a vector of systolic blood pressure, diastolic blood pressure or heart rate readings recorded as integers:

```
ll_bp_gamma = function(par, x) {
  shape = exp(par[1])
  rate = exp(par[2])
  p_round = plogis(par[3])

  liki = log( p_round * (x %% 10 == 0) *
                   (pgamma(x+4.5, shape=shape, rate=rate) -
                      pgamma(x-5.5, shape=shape, rate=rate)) +
              (1-p_round)*
                   (pgamma(x+0.5, shape=shape, rate=rate) -
                      pgamma(x-0.5, shape=shape, rate=rate) )
               )
  return (-sum(liki))
}

o = optim(par=c(log(36),log(0.3),-3), ll_bp_gamma, x=df$bp)
exp(o$par[1])
exp(o$par[2])
plogis(o$par[3])
```

Likelihood function for respiratory rate (normal distribution). Generate maximum likelihood estimates and return estimates on original scale, where df$rr is a vector of respiratory rate readings recorded as integers:

```
ll_rr = function(par, x) {
  mean = exp(par[1])
  sd = exp(par[2])
  p_round2 = plogis(par[3])
  p_round4 = plogis(par[4])

  liki = log(
        p_round2 * (x %% 2 == 0) *
               (pnorm(x+1, mean, sd) - pnorm(x-1, mean, sd)) +
         p_round4 * (x %% 4 == 0) *
               (pnorm(x+2, mean, sd) - pnorm(x-2, mean, sd)) +
         (1-p_round2-p_round4) *
               (pnorm(x+0.5, mean, sd) - pnorm(x-0.5, mean, sd))
      )
  return (-sum(liki))
}

o = optim(par=c(log(20), log(2), qlogis(0.1), qlogis(0.1)), ll_rr, x=df$rr)
exp(o$par[1])
exp(o$par[2])
plogis(o$par[3])
plogis(o$par[4])
```

Note that rounding is done at the time the observation is originally recorded, e.g. by timing for 15 seconds and multiplying by 4 or by timing for 30 seconds and multiplying by 2.

Likelihood function for respiratory rate (gamma distribution). Generate maximum likelihood estimates and return estimates on original scale, where df$rr is a vector of respiratory rate readings recorded as integers:

```
ll_rr_gamma = function(par, x) {
  shape = exp(par[1])
  rate = exp(par[2])
  p_round2 = plogis(par[3])
  p_round4 = plogis(par[4])

  liki = log(
        p_round2 * (x %% 2 == 0) *
                (pgamma(x+1, shape=shape, rate=rate) -
                       pgamma(x-1, shape=shape, rate=rate)) +
        p_round4 * (x %% 4 == 0) *
                (pgamma(x+2, shape=shape, rate=rate) -
                       pgamma(x-2, shape=shape, rate=rate)) +
        (1 - p_round2 - p_round4) *
                (pgamma(x+0.5, shape=shape, rate=rate) -
                       pgamma(x-0.5, shape=shape, rate=rate))
        )
  return (-sum(liki))
}

o = optim(par=c(log(20), log(2), qlogis(0.1), qlogis(0.1)),
        ll_rr_gamma, x=df$rr)
exp(o$par[1])
exp(o$par[2])
plogis(o$par[3])
plogis(o$par[4])
```

Supplementary figures

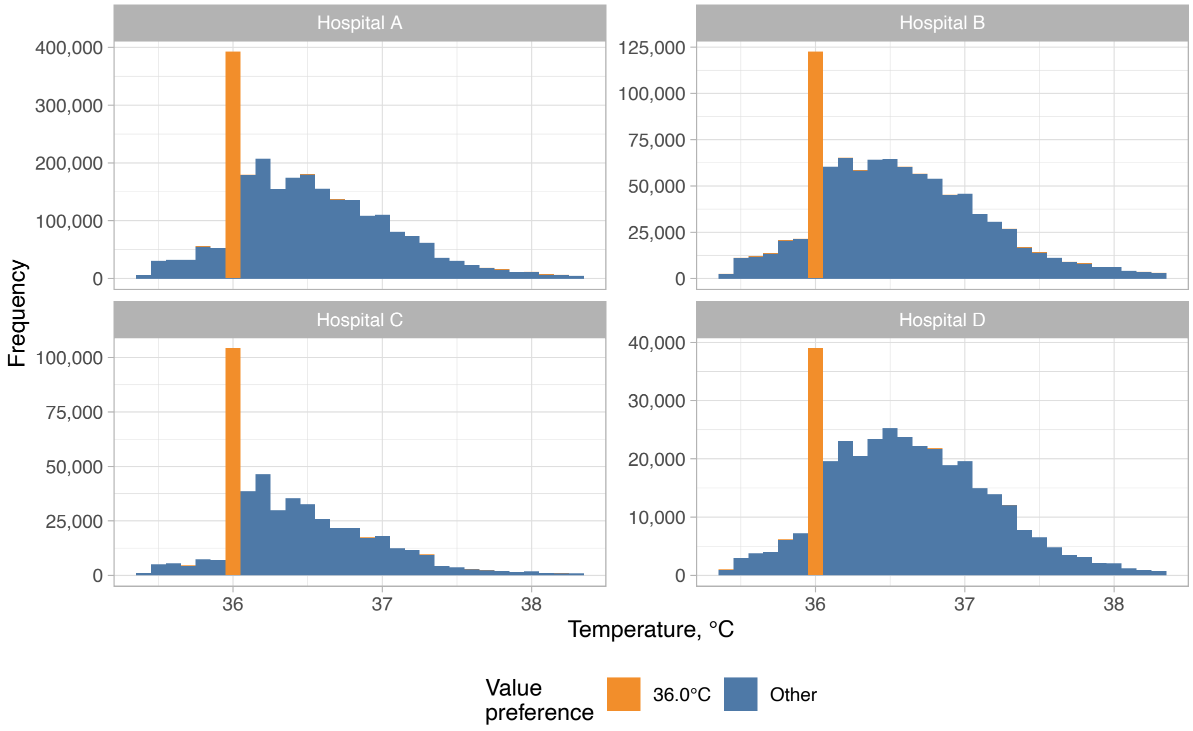

Fig S1. Observed distribution of temperature recordings, by hospital. Readings showing potential value preferences are shown in orange/red. Values below the 1st percentile or above the 99th percentile are omitted for visualisation purposes. Hospital A provides acute care, trauma, and neurosurgery services, hospital B provides elective cancer surgery, transplant, haematology, oncology services, hospital C is a district hospital providing acute care and hospital D is an elective orthopaedic hospital.

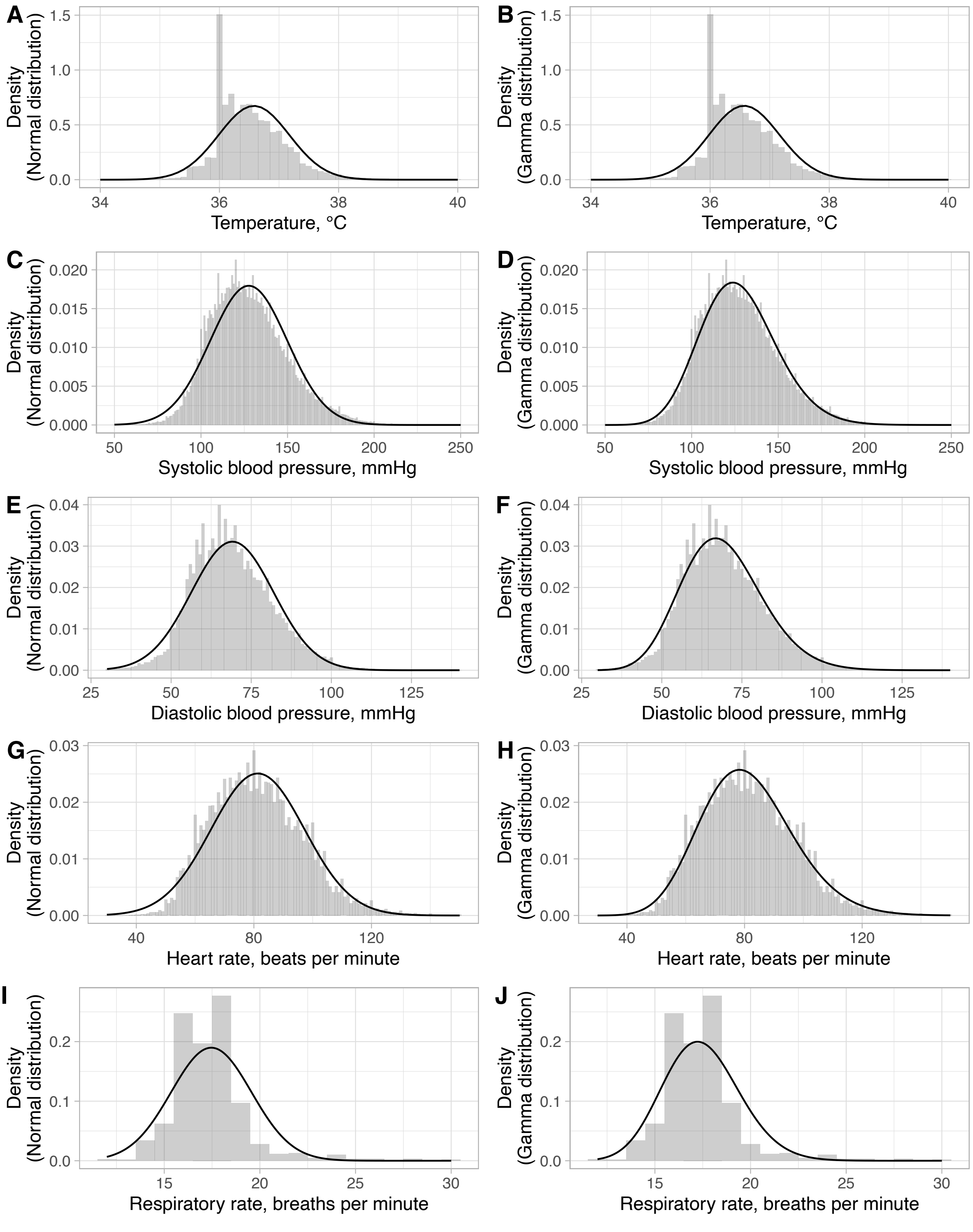

Fig S2. Observed and fitted distribution of temperature, blood pressure, heart rate, and respiratory rate measurements. The observed distribution is shown in the grey histogram and the fitted distribution as the black line. The left hand column shows fitted normal distributions and the right hand column fitted gamma distributions. Estimated value preference proportions are shown in Table 1 (normal distribution) and Table S2 (gamma distribution).

Supplementary tables

Characteristic	Data complete<br>N = 4,375,654	Missing ≥1 vital sign<br>N = 631,996
Age	71 (55, 82)	69 (53, 81)
Sex		
F	2,175,765 (87%)	322,502 (13%)
M	2,199,889 (88%)	309,494 (12%)
Hospital		
A	2,560,116 (87%)	394,510 (13%)
B	972,792 (89%)	121,623 (11%)
C	482,154 (92%)	41,599 (7.9%)
D	360,592 (83%)	74,264 (17%)
IMD percentile	74 (54, 89)	73 (53, 89)
Unknown	41,350	6,139
Ethnic group		
White	3,446,427 (87%)	493,543 (13%)
Asian	94,247 (87%)	14,528 (13%)
Black	47,522 (88%)	6,777 (12%)
Mixed	24,500 (87%)	3,686 (13%)
Other	30,177 (87%)	4,494 (13%)
Not stated or unknown	732,781 (87%)	108,968 (13%)
Charlson score	4 (0, 14)	4 (0, 13)
Day of admission		
<1 day	923,818 (83%)	190,541 (17%)
1 day	537,365 (86%)	85,973 (14%)
2 days	384,726 (88%)	54,779 (12%)
3 days	299,953 (88%)	40,490 (12%)
4-6 days	617,932 (89%)	76,803 (11%)
7-13 days	714,471 (90%)	82,468 (10%)
≥14 days	897,389 (90%)	100,942 (10%)
Hour of day		
0-1	134,039 (80%)	33,164 (20%)
2-3	106,243 (79%)	29,052 (21%)
4-5	271,196 (90%)	29,828 (9.9%)
6-7	716,831 (90%)	76,601 (9.7%)
8-9	253,741 (87%)	39,163 (13%)
10-11	553,289 (88%)	72,142 (12%)
12-13	257,292 (82%)	56,969 (18%)
14-15	366,866 (85%)	65,123 (15%)
16-17	455,512 (86%)	71,217 (14%)
18-19	285,257 (82%)	64,447 (18%)
20-21	733,477 (93%)	55,451 (7.0%)
22-23	241,911 (86%)	38,839 (14%)
Day of the week		
Monday	588,079 (89%)	72,314 (11%)
Tuesday	616,816 (88%)	86,336 (12%)
Wednesday	637,162 (86%)	99,576 (14%)
Thursday	641,550 (86%)	101,046 (14%)
Friday	648,434 (87%)	99,960 (13%)
Saturday	640,241 (87%)	94,684 (13%)
Sunday	603,372 (89%)	78,080 (11%)
Years since study start		

<1	516,696 (87%)	76,861 (13%)
1	1,324,311 (89%)	170,208 (11%)
2	1,270,394 (88%)	167,714 (12%)
3	1,264,253 (85%)	217,213 (15%)
Specialty group		
Acute & emergency medicine	660,530 (88%)	87,782 (12%)
General surgery	882,956 (89%)	112,159 (11%)
Cardiothoracic surgery	108,236 (87%)	16,185 (13%)
ENT, plastic surgery, maxillofacial surgery, ophthalmology	158,749 (88%)	21,694 (12%)
Neurosurgery	209,059 (84%)	40,213 (16%)
Trauma & orthopaedics	597,834 (87%)	91,777 (13%)
Obstetrics & gynaecology	103,948 (78%)	28,912 (22%)
Cardiology	192,020 (81%)	46,272 (19%)
Gastroenterology	129,021 (89%)	15,801 (11%)
Geratology	459,705 (88%)	61,573 (12%)
Infectious diseases	229,888 (89%)	29,438 (11%)
Nephrology	87,162 (91%)	8,324 (8.7%)
Haematology & oncology	296,195 (91%)	30,973 (9.5%)
Respiratory medicine	70,473 (90%)	7,550 (9.7%)
Other	189,878 (85%)	33,343 (15%)

Table S1. Data completeness. ENT, ear, nose and throat surgery. Data are summarised as median (interquartile range) or n (%). Hospital A provides acute care, trauma, and neurosurgery services, hospital B provides elective cancer surgery, transplant, haematology, oncology services, hospital C is a district hospital providing acute care and hospital D is an elective orthopaedic hospital.

Vital sign	Estimated value preference proportion (95% CI)	Estimated underlying distribution parameters (95% CI)
**Temperature**<br>Excess of readings of 36.0°C	11.3% (10.6-12.0%)	Shape: 3824 (3639-3985)<br>Rate: 105 (99-109)
**Systolic blood pressure**<br>Readings subject to rounding to nearest 10	2.1% (1.4-2.9%)	Shape: 34 (33-35)<br>Rate 0.26 (0.26-0.27)
**Diastolic blood pressure**<br>Readings subject to rounding to nearest 10	2.0% (1.2-2.7%)	Shape: 30 (29-31)<br>Rate: 0.43 (0.42-0.44)
**Heart rate**<br>Readings subject to rounding to nearest 10	2.4% (1.7-3.1%)	Shape: 27 (26-27)<br>Rate: 0.33 (0.32-0.34)
**Respiratory rate**<br>Readings subject to rounding to multiples of 2 or 4	Multiple of 2: 24.7% (21.5-26.8%)<br>Multiple of 4: <0.1% (<0.1-0.1%)	Shape: 75 (71-82)<br>Rate: 4.3 (4.1-4.7)

Table S2. Estimated value preference proportions and underlying distributions for temperature, blood pressure, heart rate, and respiratory rate assuming an underlying gamma distribution. See Table 1 for estimates assuming an underlying normal distribution.

	Temperature			Systolic and diastolic blood pressure			Heart rate			Respiratory rate		
Characteristic	aOR	95% CI	p-value	aOR	95% CI	p-value	aOR	95% CI	p-value	aOR[1]	95% CI	p-value
Specialty group												
Acute & emergency medicine	—	—		—	—		—	—		—	—	
General surgery	0.85	0.83, 0.87	<0.001	0.65	0.61, 0.68	<0.001	0.95	0.93, 0.96	<0.001	1.10	1.09, 1.11	<0.001
Cardiothoracic surgery	0.92	0.89, 0.96	<0.001	0.79	0.74, 0.84	<0.001	1.01	0.98, 1.04	0.65	1.12	1.10, 1.15	<0.001
ENT, plastic surgery, maxillofacial surgery, ophthalmology	1.25	1.21, 1.29	<0.001	0.77	0.72, 0.82	<0.001	1.01	0.97, 1.04	0.75	0.99	0.97, 1.00	0.092
Neurosurgery	0.97	0.94, 1.00	0.10	0.65	0.62, 0.69	<0.001	0.95	0.93, 0.97	<0.001	1.23	1.21, 1.25	<0.001
Trauma & orthopaedics	0.79	0.77, 0.81	<0.001	0.78	0.75, 0.81	<0.001	0.96	0.94, 0.98	<0.001	0.96	0.95, 0.98	<0.001
Obstetrics & gynaecology	0.69	0.66, 0.72	<0.001	0.63	0.58, 0.68	<0.001	0.96	0.93, 0.98	<0.001	1.25	1.23, 1.27	<0.001
Cardiology	0.87	0.85, 0.89	<0.001	0.81	0.77, 0.85	<0.001	1.07	1.04, 1.09	<0.001	1.00	0.99, 1.01	0.88
Gastroenterology	0.95	0.92, 0.98	0.002	0.86	0.80, 0.92	<0.001	0.97	0.94, 0.99	0.004	1.23	1.21, 1.25	<0.001
Geratology	0.96	0.95, 0.98	<0.001	1.11	1.07, 1.15	<0.001	1.00	0.98, 1.01	0.68	1.04	1.03, 1.05	<0.001
Infectious diseases	0.93	0.91, 0.95	<0.001	0.96	0.90, 1.02	0.14	1.00	0.98, 1.02	>0.99	1.03	1.02, 1.04	<0.001
Nephrology	0.79	0.75, 0.83	<0.001	0.74	0.68, 0.82	<0.001	0.94	0.91, 0.98	<0.001	0.98	0.96, 1.00	0.033
Haematology & oncology	0.69	0.66, 0.72	<0.001	5.78	5.32, 6.28	<0.001	1.05	1.03, 1.07	<0.001	1.23	1.21, 1.25	<0.001
Respiratory medicine	1.16	1.10, 1.22	<0.001	0.97	0.89, 1.05	0.42	1.05	1.02, 1.08	0.001	0.79	0.77, 0.81	<0.001
Other	0.93	0.91, 0.96	<0.001	0.88	0.83, 0.93	<0.001	0.98	0.96, 1.00	0.044	1.08	1.06, 1.09	<0.001

Table S3. Multivariable relationships between value preferences in temperature, blood pressure, heart rate and respiratory rate and specialty caring for patients. aOR = adjusted odds ratio, CI = confidence interval. Estimates are also plotted in Figure 3. Other factors adjusted for are shown in Table 3 and Figure 2.
